# Uptake of COVID-19 vaccination in a medium secure psychiatric hospital population

**DOI:** 10.1192/bjo.2021.924

**Published:** 2021-06-01

**Authors:** Simon Gibbon, Emma McPhail, Georgina Mills, Martin McBride, Rebekah Storer, Nicholas Taylor, Lucy McCarthy

**Affiliations:** East Midlands Centre for Forensic Mental Health, Nottinghamshire Healthcare NHS Foundation Trust, Arnold Lodge, UK

**Keywords:** Consent and capacity, forensic mental health services, epidemiology, comorbidity, in-patient treatment

## Abstract

Patients in medium secure hospitals may be at particularly increased risk of coronavirus disease 2019 (COVID-19) infection and complications. We undertook a service evaluation involving all current in-patients within a single, English medium secure hospital to describe the uptake of the COVID-19 vaccine among this population. Data regarding capacity to consent to the vaccine, acceptance/refusal of this (and reasons for refusal) and demographics was retrospectively collected from the patients’ clinical records and analysed. In total, 85 patients (92.4% of eligible patients) had capacity to decide if they wanted the COVID-19 vaccine. Of these 68 (80.0%) consented and 17 (20.0%) declined to consent. A similar proportion of patients aged under and over 40 years old consented to have the vaccine. Those from a Black Asian minority ethnic background were more likely to decline the vaccine than White British patients. The reasons for capacitous refusal appeared similar to those seen in the general population.

## Background

Compared with the general population, people with mental health disorders are at increased risk of negative physical and mental health outcomes (including increased mortality) following SARS-CoV-2 infection.^1,2^ Those in longer-term care institutions may also be more exposed to infection via frequent contact with other patients and staff in their care setting.^3^ Mental health in-patients (including those with severe mental illness and intellectual impairment) may thus be particularly vulnerable in the current coronavirus disease 2019 (COVID-19) pandemic. In the UK (at the time of writing in April 2021), all adult mental health in-patients are being offered the COVID-19 vaccine as they can be considered to be in ‘Category 6’ as defined by the Joint Committee on Vaccination and Immunisation (JCVI) advice on priority groups for COVID-19 vaccination.^4^

Factors that may promote or inhibit uptake of COVID-19 vaccine in the general population (such as prosocial beliefs in the collective importance of taking the vaccine; vaccine efficacy; side-effects; speed of development of COVID-19 vaccines; or mistrust) are beginning to be elucidated,^5^ however, little is known about the vaccine uptake in people with mental disorders. We are not aware of any studies on COVID-19 vaccination uptake involving mental health patients detained in secure hospital settings.

Patients admitted to medium secure care have greatly increased mortality compared with the general population.^6^ Most are admitted from prison and have a diagnosis of severe mental illness often complicated by histories of offending, substance misuse and trauma.^7^ Understanding COVID-19 vaccine uptake, and reasons for refusal, in patients in medium secure hospitals is especially important given the high prevalence of chronic physical health comorbidities such as obesity and diabetes seen in this population,^8^ as these conditions are also associated with poor clinical outcomes in severe COVID-19 disease.^9^

## Aims

We therefore undertook a rapid service evaluation within the medium secure hospital in which we work to examine the proportions of patients who accepted or declined to accept the COVID-19 vaccine, and explored the reasons for patients’ refusal of the vaccine. Given the findings in the general population, we were particularly keen to examine: if there was a difference in vaccine uptake between White and Black Asian minority ethnic (BAME) patients and if older patients were more likely to accept the vaccine than younger patients.

## Method

The study took place at Arnold Lodge in Leicester, UK. This is a medium secure hospital that provides care for adult males and females. It was conducted as a service evaluation with local permission from the Nottinghamshire Healthcare NHS Foundation Trust Research and Evidence department. As per the JCVI guidance, all patients in the hospital were offered a COVID-19 vaccine. As part of this this they all had a capacity assessment and physical health evaluation completed by their consultant forensic psychiatrist.

Data on the outcome of these assessments together with demographic data was collected from the patients’ electronic notes, anonymised and entered onto an electronic database for analysis. Descriptive statistics were reported for ‘eligibility to receive the vaccine’, age, gender, ethnicity (BAME or White British) and JCVI status.

## Results

Of the 96 in-patients, 4 were ineligible for the vaccine because of recently having a COVID-19 infection. There was no statistically significant difference in the mean age of the 72 male and 20 female patients who comprised the group of 92 patients eligible to receive the vaccine (overall mean age 38.7 years; age range 19–62 years). A quarter (23/92) of the eligible patients were BAME and three-quarters were White British. Three patients were assessed as being in ‘Category 4’ of the JCVI system (i.e. those 70 years of age and over or clinically extremely vulnerable individuals) and the remainder were in ‘Category 6’ by reason of their serious mental illness.

Of the 92 patients who were eligible, 7 patients (7.6%) were considered by their consultant forensic psychiatrist to lack capacity to make the decision regarding the vaccine. For two of these patients it was judged by their clinical team to be in their best interests to be given the vaccine; for the other five patients, the clinical team judged it not to be in the patients’ best interests because they would require physical restraint which would cause disproportionate distress and have a negative impact upon therapeutic relationships.

In total, 85 patients (92.4% of eligible patients) had capacity to decide if they wanted the COVID-19 vaccine. Of these 68 (80.0%) consented and 17 (20.0%) declined to consent. A similar proportion of patients aged under and over 40 years old (40/51, 78.4% *v.* 28/34, 82.4.4%) consented to have the vaccine. Those from a BAME background were more likely to decline the vaccine than White British patients (6/20, 30% *v.* 11/65, 16.9%).

Of those patients who gave capacitous refusal to have the vaccine (*n* = 17), the most commonly cited reasons were concerns about the safety profile/side-effects of the vaccine (*n* = 5, 29.4%) and patients’ perception of having a low risk to their personal health from COVID-19 (*n* = 4, 23.5%). Five patients were unwilling to explain their decision, two cited broader distrust of healthcare services and one had concerns about animal testing.

## Discussion

This small-scale study in a single site shows that among a group of patients who are detained in a medium secure hospital setting, immunisation with the COVID vaccine was broadly acceptable and most patients gave consent to receive it. The latest overall NHS England statistics show that 76.9% of individuals identified as being in an at-risk group or who are an unpaid carer have had at least one dose of a vaccine,^10^ so the prevalence of capacitous refusal in our sample appears similar to that seen in the general English population.^10^ Some of our patients’ explanations for refusing a vaccine (e.g. concerns about the safety/side-effects; general distrust) have also been identified as factors associated with hesitancy in vaccine uptake in the community.^5^

Although we were able to collect data from all in-patients at our medium secure hospital the relatively small number of participants and single site nature of this study limits the generalisability of our findings and prevented us from doing adequate powered statistical analysis of between-group differences.

The indication that patients of BAME ethnicity were more likely than White patients to decline the vaccination echoes the findings of research conducted in the general hospital trust that serves our city (Leicester) which showed a reduced uptake of the vaccine in South Asian or Black staff compared with White staff (uptake rates 59%, 37%, 71% respectively).^11^ Further consideration needs to be given to how the uptake of COVID-19 vaccination can be improved in people with BAME ethnicity, especially as this group is also over-represented in secure hospital settings.^12^

We acknowledge that the study has clear limitations as it describes the acceptability of a COVID-19 vaccine in a single secure hospital population in the UK. Nonetheless, the study demonstrates that similar services caring for people with severe mental disorders should be able to approach the vaccination process with confidence. As many people with severe mental disorder also have high levels of physical health conditions such as obesity, diabetes and cardiovascular problems that would increase the risk of a poor clinical outcome if they contract COVID-19, protecting this vulnerable population through vaccination must be a priority for all mental health services.

## Data Availability

The data that support the findings of this study are available on request from the corresponding author, S.G.

## References

[1] Zhang S, Das-Munshi J, Thornicroft G. Safeguarding the physical health of people with severe mental disorders during the COVID-19 pandemic. BJPsych Bull 2020; 44: 223–4. 32981560 10.1192/bjb.2020.92 PMC7525586

[2] Jeon HL, Kwon JS, Park SH, Shin JY. Association of mental disorders with SARS-CoV-2 infection and severe health outcomes: nationwide cohort study. Br J Psychiatry [Epub ahead of print] 7 Jan 2021. Available from: 10.1192/bjp.2020.251. 33407954

[3] Pineles L, Perencevich EN, Roghmann MC, Gupta K, Cadena J, Baracco G, Frequency of nursing home resident contact with staff, other residents, and the environment outside resident rooms. Infect Control Hosp Epidemiol 2019; 40: 815–6. 31106727 10.1017/ice.2019.117 PMC7191993

[4] Department of Health and Social Care, UK. Joint Committee on Vaccination and Immunisation: Advice on Priority Groups for COVID-10 Vaccination. Department of Health and Social Care, UK, 2020 (https://www.gov.uk/government/publications/priority-groups-for-coronavirus-covid-19-vaccination-advice-from-the-jcvi-30-december-2020/joint-committee-on-vaccination-and-immunisation-advice-on-priority-groups-for-covid-19-vaccination-30-december-2020).

[5] Freeman D, Loe BS, Chadwick A, Vaccari C, Waite F, Rosebrock L, COVID-19 vaccine hesitancy in the UK: the Oxford coronavirus explanations, attitudes, and narratives survey (Oceans) II. Psychol Med [Epub ahead of print] 11 Dec 2021. Available from: 10.1017/S0033291720005188. PMC7804077 33305716

[6] Davies S, Clarke M, Hollin C, Duggan C. Long-term outcomes after discharge from medium secure care: a cause for concern. Br J Psychiatry 2007; 191: 70–4. 17602128 10.1192/bjp.bp.106.029215

[7] Gibbon S, Huband N, Bujkiewicz S, Hollin CR, Clarke M, Davies S, The influence of admission characteristics on outcome: evidence from a medium secure forensic cohort. J Pers Ment Health 2013; 7: 1–10. 10.1002/pmh.1191 24343920

[8] Public Health England. Working Together to Address Obesity in Adult Mental Health Secure Units. Public Health England, 2016 (https://www.gov.uk/government/uploads/system/uploads/attachment_data/file/591875/obesity_in_mental_health_secure_units.pdf).

[9] Barron E, Bakha C, Kar P, Weaver A, Bradley D, Ismail H, Associations of type 1 and type 2 diabetes with COVID-19-related mortality in England: a whole-population study. Lancet Diabetes Endocrinol 2020; 8: 813–22. 32798472 10.1016/S2213-8587(20)30272-2 PMC7426088

[10] NHS England, UK. *COVID-19 Weekly Announced Vaccinations, 2021.* NHS England, 2021 (https://www.england.nhs.uk/statistics/statistical-work-areas/covid-19-vaccinations/).

[11] Iacobucci G. Covid:19: ethnic minority health staff are less likely to take up vaccine, early data show. BMJ 2021; 372: n460. 33593814 10.1136/bmj.n460

[12] Coid J, Kahtan N, Gault S, Jarman B, Ethnic differences in admissions to secure forensic psychiatry differences in admissions to secure forensic psychiatry services. Br J Psychiatry 2000; 17: 241–7. 10.1192/bjp.177.3.241 11040885

